# ASO Author Reflections: Arterial Ischemic Events Following Cancer Surgery: Where Do We Stand?

**DOI:** 10.1245/s10434-023-14816-2

**Published:** 2024-01-11

**Authors:** Juhana Rautiola, Johan Björklund, Renata Zelic, Gustaf Edgren, Matteo Bottai, Magnus Nilsson, Per Henrik Vincent, Hanna Fredholm, Henrik Falconer, Annika Sjövall, Per J. Nilsson, Peter Wiklund, Markus Aly, Olof Akre

**Affiliations:** 1 Department of Molecular Medicine and Surgery, Karolinska Institutet and Department of Pelvic Cancer, Karolinska University Hospital, Stockholm, Sweden; 2 Clinical Epidemiology Division, Department of Medicine, Karolinska Institutet and Department of Cardiology, Södersjukhuset, Stockholm, Sweden; 3 Division of Biostatistics, Institute of Environmental Medicine, Karolinska Institutet, Stockholm, Sweden; 4 Division of Surgery and Oncology, Department of Clinical Science, Intervention and Technology, Karolinska Institutet and Department of Upper Abdominal Diseases, Karolinska University Hospital, Stockholm, Sweden; 5 Department of Molecular Medicine and Surgery, Karolinska Institutet, Stockholm, Sweden; 6 Department of Pelvic Cancer, Karolinska University Hospital and Department of Women’s and Children’s Health, Karolinska Institutet, Stockholm, Sweden; 7 Department of Urology, Icahn School of Medicine at Mount Sinai, New York, NY USA

An elevated risk of arterial ischemic events following non-cardiac surgery has been demonstrated in various studies,^1^ as has the association of elevated troponin levels with 30-day mortality.^2^ Additionally, cancer patients face an augmented risk of cardiovascular complications, likely attributable to treatments associated with their disease.^3^ There is a lack of data on the risk of cardiovascular complications following surgery for different cancers. The aim of this observational cohort study was to improve pre- and postoperative management of cardiovascular complications, tailored for different cancers.

At present, risk assessment is performed routinely based on background comorbidities without taking tumor type into consideration. In this article, we show that different tumor types possess different risk for myocardial infarction or ischemic stroke. Furthermore, we show that in certain tumors, the transient increase of risk is short, up to 1 week after surgery, whereas in some cancers the risk persists over a longer time.^4^

In the future, prospective evaluation of preoperative risk factors is needed to enable selection of patients who need more comprehensive cardiovascular risk assessment before surgery, including intervention. Additionally, it is essential to evaluate the protective effects of antithrombotic medication on outcome incidences.
